# Cells: Are They (Still) Essential for Dental Regeneration?

**DOI:** 10.3390/cells10030498

**Published:** 2021-02-26

**Authors:** Marco Tatullo, Maria Giovanna Gandolfi

**Affiliations:** 1 Department of Basic Medical Sciences, Neurosciences and Sense Organs, University of Bari Aldo Moro, 70124 Bari, Italy; 2 Marrelli Health—Tecnologica Research Institute, Tecnologica Research Institute, Stem Cell Unit, 88900 Crotone, Italy; 3 Laboratory of Biomaterials and Oral Pathology, Department of Biomedical and Neuromotor Sciences, School of Dentistry, University of Bologna, 40125 Bologna, Italy; mgiovanna.gandolfi@unibo.it

Tissue regeneration in dentistry has demonstrated impressive progress over during the last decades compared to other medical sciences.

The modern concept of oral stem cells emerged in the late 1990s, mainly targeting cell-based therapy with in vitro prepared cells delivered into the host to regenerate tissue in large defects, which is unachievable via the cell-free approach [1]. The scientific community has started an exciting debate on the pros and cons of categorizing regenerative dentistry into the cell-based (CBA) approach and cell-free approach (CFA).

However, while this debate is worthy of great attention, it is considerably difficult to make a clear choice between CBA and CFA, as the clinical outcomes may typically involve several aspects related to local cell biology, tissue engineering and surgical procedures [2,3].

An interesting paper attempted to determine the main differences among stem cell-based and molecular-based approaches in such regenerative procedures applied to dental sciences [4]. Of course, the molecular aspects greatly influence cell behavior and clinical healing; as an example, the expression of surface marker CD146 has been demonstrated to significantly influence [5] the properties of mesenchymal stem cells (MSCs) derived from the inflammatory human periapical cystic wall, called human periapical cyst–mesenchymal stem cells (hPCy-MSCs) [6]. Nevertheless, in regenerative dentistry, we should consider the influence of the local environment on stem cell-based regeneration: to overcome the CBA, a promising strategy may be to involve the glycogen synthase kinase-3 (GSK-3) antagonists, which have been linked to the activity of Wnt signaling and to the regeneration of injured tissues, including complex dental and oral structures. Such class of molecules seems to work with a kind of “bystander effect”; in fact, they can modulate local inflammation and bone resorption, triggering differentiation of resident “sleeping” MSCs and promoting the homing of circulating MSCs on site [7].

Recently, the use of microvesicles (MVs) obtained from cultured dental-derived MSCs [8] has gained the interest of several researchers; the most suitable MVs are exosomes, which are simply obtained from MSC conditioned medium, and may also be easily stored for clinical purposes, drastically reducing the ethical issues related to cell-based therapy [9].

In conclusion, there is a general consensus on the need for more rigorous basic research to definitively endorse and clinically validate a CFA in dental regeneration. Nonetheless, it is notable that several doubts and pitfalls still affect the concept of regenerative dentistry [10]; in this context, it is recommended to conduct more trials on CFAs compared to CBAs, strengthening the methodology of translational research on dental stem cell biology and oral tissue engineering. In the future, we may be able to regenerate dental tissues without manipulating cells; we are all a small but significant part of this big challenge.
